# *Rumex japonicus* Houtt. Extract Suppresses Colitis-Associated Colorectal Cancer by Regulating Inflammation and Tight-Junction Integrity in Mice

**DOI:** 10.3389/fphar.2022.946909

**Published:** 2022-07-05

**Authors:** Hee-Young Kim, Ji Eun Seo, Hanul Lee, Chang-Hwan Bae, Ki-Tae Ha, Seungtae Kim

**Affiliations:** ^1^ Korean Medicine Research Center for Healthy Aging, Pusan National University, Yangsan, South Korea; ^2^ Department of Korean Medical Science, School of Korean Medicine, Pusan National University, Yangsan, South Korea

**Keywords:** azoxymethane, colon cancer, dextran sulfate sodium, Rumex japonicus houtt, tight junction

## Abstract

Irritable bowel disease (IBD), which results in an elevated risk of colitis-associated colorectal cancer (CAC), is characterized by inflammation and barrier disruption of the gut. The genus *Rumex* has anti-oxidative and anti-inflammatory effects, and the roots of *Rumex japonicus* Houtt (RJ) have been traditionally used in East Asia to treat digestive problems. We investigated the protective effect of RJ against azoxymethane (AOM)-and dextran sulfate sodium (DSS)-induced CAC in C57BL/6N male mice. The mice were intraperitoneally injected with AOM on the first day and orally treated with 2% DSS for 2 weeks (on the third and sixth weeks). RJ extract (100 mg/kg) was administered to the mice in the RJ group for 4 weeks (from the third to sixth week), and all mice were sacrificed on the final day of the eighth week. Changes in morphology, tight junctions (TJs), inflammation-related factors in the colon and serum inflammatory cytokine levels were measured. The colons of AOM/DSS-treated mice were shorter and heavier than those of normal mice. The number of tumors in the colons of AOM/DSS-treated mice increased; however, RJ suppressed these changes. RJ also reduced the levels of tumor necrosis factor-α, interleukin (IL)-6, and IL-1β in the colon and serum, and it increased the level of IL-10 in the colon. Moreover, RJ inhibited the barrier disruption and apoptosis in the colons of AOM/DSS-treated mice. RJ effectively suppressed AOM/DSS-induced CAC by inhibiting tumor formation, inflammation, disruption of TJ, and apoptosis in the colon.

## Introduction

Colorectal cancer is the third most common cancer worldwide, and its prevalence is expected to rise by 60% by 2030 (Ferlay et al., 2013). Inflammatory bowel diseases (IBD), including Crohn’s disease and ulcerative colitis (UC), are chronic multifactorial gastrointestinal inflammatory disorders characterized by chronic inflammatory reaction, epithelial barrier dysfunction, diarrhea, abdominal pain, and enterohemorrhage (Zhu et al., 2019). Though the pathogenesis and etiology of IBD are not clear, dysregulation of the gut barrier and mucosal immune system may be involved in the pathogenesis (Round and Mazmanian, 2009). According to a meta-analysis, the incidence of colorectal cancer increased as the onset period of UC increased (Eaden et al., 2001). The increase in mortality and morbidity of IBD is related to the incidence of colitis-associated colorectal cancer (CAC) (Ekbom et al., 1990). Therefore, chronic IBD is an important risk factor for CAC (Shih and Targan, 2008).

*Rumex japonicus* Houtt (RJ) is widely distributed in East Asia. RJ is a perennial herb, and the root of RJ has been traditionally used to treat various diseases, such as constipation, jaundice, uterine bleeding, and hematemesis (Sun et al., 2020). RJ has anti-oxidative, anti-bacterial, anti-sepsis, anti-inflammatory, and anti-cancer effects (Sun et al., 2020). Moreover, RJ alleviates the disruption of tight junctions (TJs), apoptosis, and inflammation in the colons of dextran sulfate sodium (DSS)-induced colitis mouse model and 1-methyl-4-phenyl-1,2,3,6-tetrahydropyridine (MPTP)-induced Parkinson’s disease mouse model (Kim et al., 2020; Kim et al., 2022). However, it is unclear whether RJ can suppress the incidence of CAC. Therefore, we investigated the protective effect of RJ in an azoxymethane (AOM)- and DSS-induced CAC mouse model.

## Materials and Methods

### Sample Preparation

RJ (grown in Gyeongsangbukdo, Korea in 2015) was purchased from Kwangmyungdang Medical Herbs (Ulsan, Korea). RJ (10 g) was immersed in 500 ml methanol, sonicated for 15 min, and extracted for 24 h. The extract was filtered through filter paper, dried using a vacuum evaporator (Eyela, Tokyo, Japan), and freeze-dried (Labconco, Kansas City, MO, United States). Constituents of RJ extract were identified by ultra-performance liquid chromatography coupled with quadrupole-time-of-flight tandem mass spectrometry (Supplementary Figure S1) (Kim et al., 2022). Freeze-dried RJ was dissolved in water for each oral administration.

### Animals

Male C57BL/6J mice (5-week-old) were obtained from Samtako Bio Korea (Osan, Korea). Mice were maintained under controlled conditions (22 ± 2°C, 55 ± 5% humidity, 12-h light/dark light cycle) with food and water ad libitum. The animal study protocol was approved by the Institutional Animal Care and Use Committee of Pusan National University (Busan, Korea; approval number PNU-2018-1847).

### Animal Study Design

**FIGURE 1:**
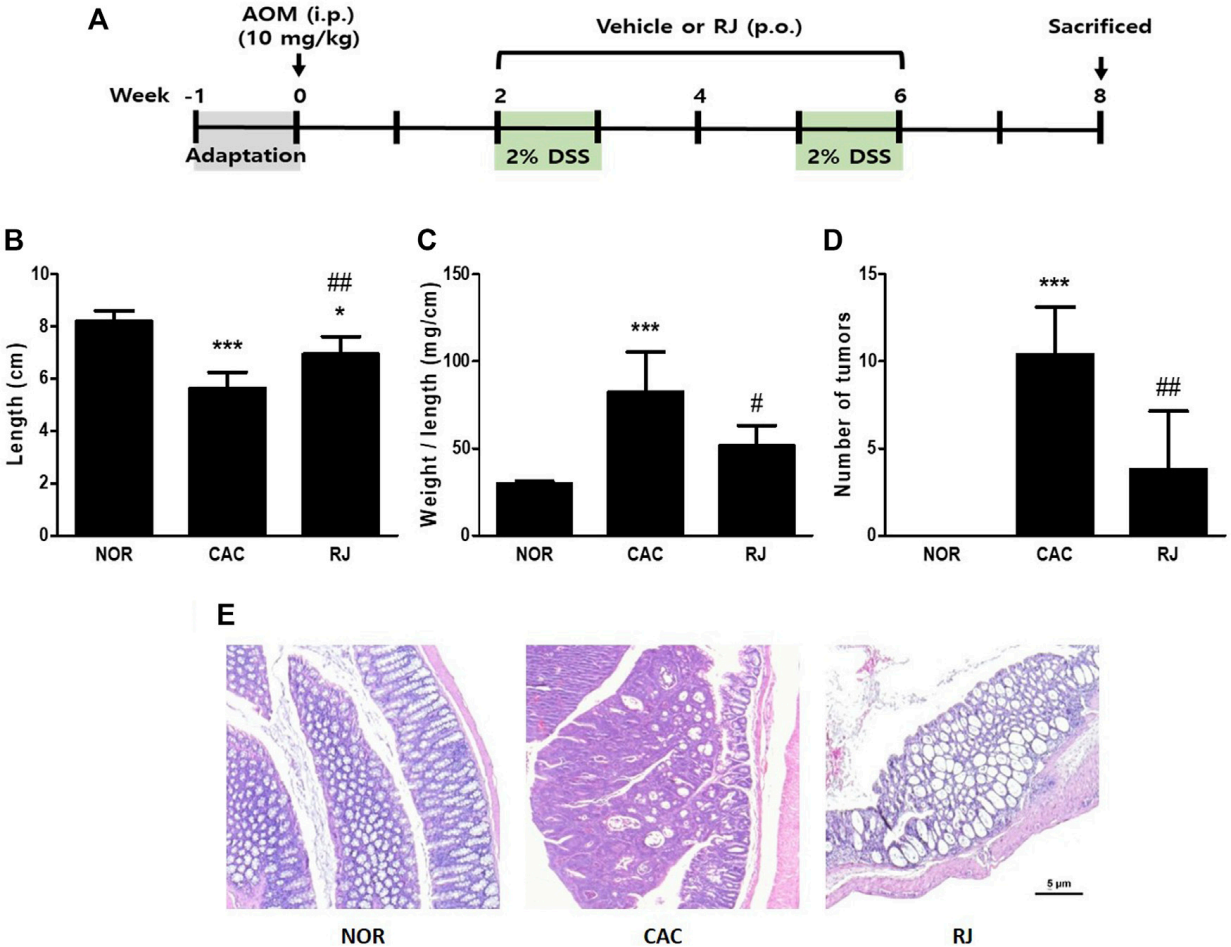
Study design and pathomorphological changes in the colons of AOM/DSS- or RJ-treated mice. Experimental procedure **(A)**. Length **(B)**, length/weight ratio **(C)**, tumor numbers **(D)**, and the histological changes **(E)** of the colon was evaluated. Values are the means ± standard deviation (*n* = 6). ^*^ *p* < 0.05 and ^***^ *p* < 0.001 versus the normal group; ^#^ *p* < 0.05 and ^##^ *p* < 0.01 versus the CAC group. AOM, azoxymethane; i.p., intraperitoneally; p.o., per oral; RJ, *Rumex japonicus* Houtt.; DSS, dextran sulfate sodium; CAC, colitis-associated colorectal cancer.

The animal study design is illustrated in Figure 1A. The mice were acclimatized for a week and then randomly divided into three groups: normal, CAC, and RJ. The CAC and RJ groups were administered a single intraperitoneal injection of AOM (10 mg/kg, Sigma-Aldrich, St. Louis, MO, United States) on the first day and 2% DSS (w/v, MP Biomedical, Solon, OH, United States) in drinking water for 2 weeks (on third and sixth week each) during the experimental period. The RJ group was administered RJ extract (100 mg/kg/day) for 4 weeks (from third week to sixth week), the dosage was determined based on our previous research (Kim et al., 2020). Meanwhile, the normal and CAC groups were administered the vehicle (water) during the same period. Finally, all mice were euthanized using isoflurane and sacrificed.

### Hematoxylin and Eosin Staining

Colon tissues were harvested, fixed in 4% (v/v) neutral-buffered formalin, and embedded in paraffin. Colon sections were stained with H&E (Sigma-Aldrich), dehydrated, and mounted. Images were acquired using an Axio Scope A1 microscope (Carl ZEISS, Oberkochen, Germany) with an AxioCam ICc3 camera (Carl ZEISS).

### Immunofluorescent Staining

Colon tissues were fixed in 4% paraformaldehyde (PFA) and soaked in 30% (w/v) sucrose solution. The tissues were embedded in cryomolds at −80°C. The 20 μm-thick colonic sections were fixed in 4% PFA for 15 min and rinsed three times with phosphate-buffered saline (PBS). The sections were then blocked using blocking buffer [PBS containing 5% (v/v) normal goat serum and 0.3% (v/v) Triton X-100] for 60 min at room temperature. The sections were treated with anti-zonula occludens (ZO)-1, anti-occludin, or anti-claudin-2 antibodies (all from Santa Cruz Biotechnology, Santa Cruz, CA, United States) overnight at 4°C. The cells or tissue sections were rinsed and treated with fluorochrome-conjugated secondary antibodies Alexa-488 or Alexa-594 IgG (Molecular Probes, Eugene, OR, United States) for 1 h at RT in the dark, dehydrated, and mounted. Immunofluorescence images were acquired using a Zeiss Axio Imager M1 microscope (Carl ZEISS).

### Western Blot

Colonic tissues were lysed with RIPA buffer (Invitrogen Life Technologies, Carlsbad, CA, United States) and centrifuged at 13,000 rpm for 15 min at 4°C. Protein concentration was determined using a Bio-Rad protein assay kit (Hercules, CA, United States). Proteins were separated by sodium dodecyl sulfate-polyacrylamide gel electrophoresis and electrotransferred to a nitrocellulose blotting membrane (GE Healthcare United Kingdom Ltd. Little Chalfont, United Kingdom). The blots were incubated with anti-tumor necrosis factor (TNF)-α, anti-cleaved caspase-3 (all from Cell Signaling Technology Inc. Beverly, MA, United States), anti-interleukin (IL)-6, anti-IL-10, anti-cyclooxygenase (COX)-2, anti-Bax (all from Abcam, Cambridge, United Kingdom), anti-IL-1β, anti-occludin, anti-ZO-1, anti-claudin-2, anti-p53, anti-p21, or anti-β-actin (all from Santa Cruz Biotechnology) primary antibodies, and immersed in 5% bovine serum albumin overnight at 4°C. The blots were washed three times with PBS containing 0.05% (v/v) Tween 20 and incubated with secondary antibodies for 1 h at RT. The membranes were then washed three times with PBS and visualized using an enhanced chemiluminescence reagent (Thermo Fisher Scientific Inc. Rochford, IL, United States).

### Enzyme-Linked Immunosorbent Assay

Mouse serum was collected from blood by centrifugation (3,000 rpm, 10 min). The changes in TNF-α, IL-6, and IL-1β were confirmed using ELISA kits (Biolegend, San Diego, CA, United States) according to the manufacturer’s protocols.

### Statistical Analysis

All experimental data are presented as the mean ± standard deviation. Differences between means were evaluated by one-way analysis of variance with Tukey’s multiple-range tests using Prism 5 (GraphPad Software Inc. La Jolla, CA, United States). Differences were considered statistically significant at *p* < 0.05.

## Results

### RJ Alleviated the Morphological Change and Tumor Formation in the Colons of the AOM/DSS-Treated Mice

The colon length of mice in the normal group was 8.2 ± 0.4 cm. The length in the CAC group was significantly shorter than that in the normal group (5.6 ± 0.5 cm, *p* < 0.001); however, the length in the RJ group was longer than that in the CAC group (6.9 ± 0.6 cm, *p* < 0.01; Figure 1B). The colon weight/length ratio in the CAC group (82.4 ± 20.7 mg/cm) was significantly higher than that in the normal group (29.8 ± 1.3 mg/cm, *p* < 0.001). However, the ratio in the RJ group (48.06 ± 8.1 mg/cm) was significantly lower than that in the CAC group (*p* < 0.05; Figure 1C). The colon tissues in the CAC group contained many tumors, but oral treatment with RJ effectively reduced tumor formation (*p* < 0.01; Figure 1D).

Histological images of H&E-stained colon tissues are shown in Figure 1E and Supplementary Figure S2. The colon tissues of the CAC group showed erosive lesions and severe dysplastic crypts. In addition, the cells in the mucous layers were severely transformed; therefore, adenomas or adenocarcinomas formed. However, the colon tissue of the RJ group showed fewer histological changes than that of the CAC group. In the RJ group, the vertical crypts were not intact goblet cells and only a few adenomas or adenocarcinomas were observed.

### RJ Reduced Colonic Inflammation in the AOM/DSS-Induced Mice

**FIGURE 2:**
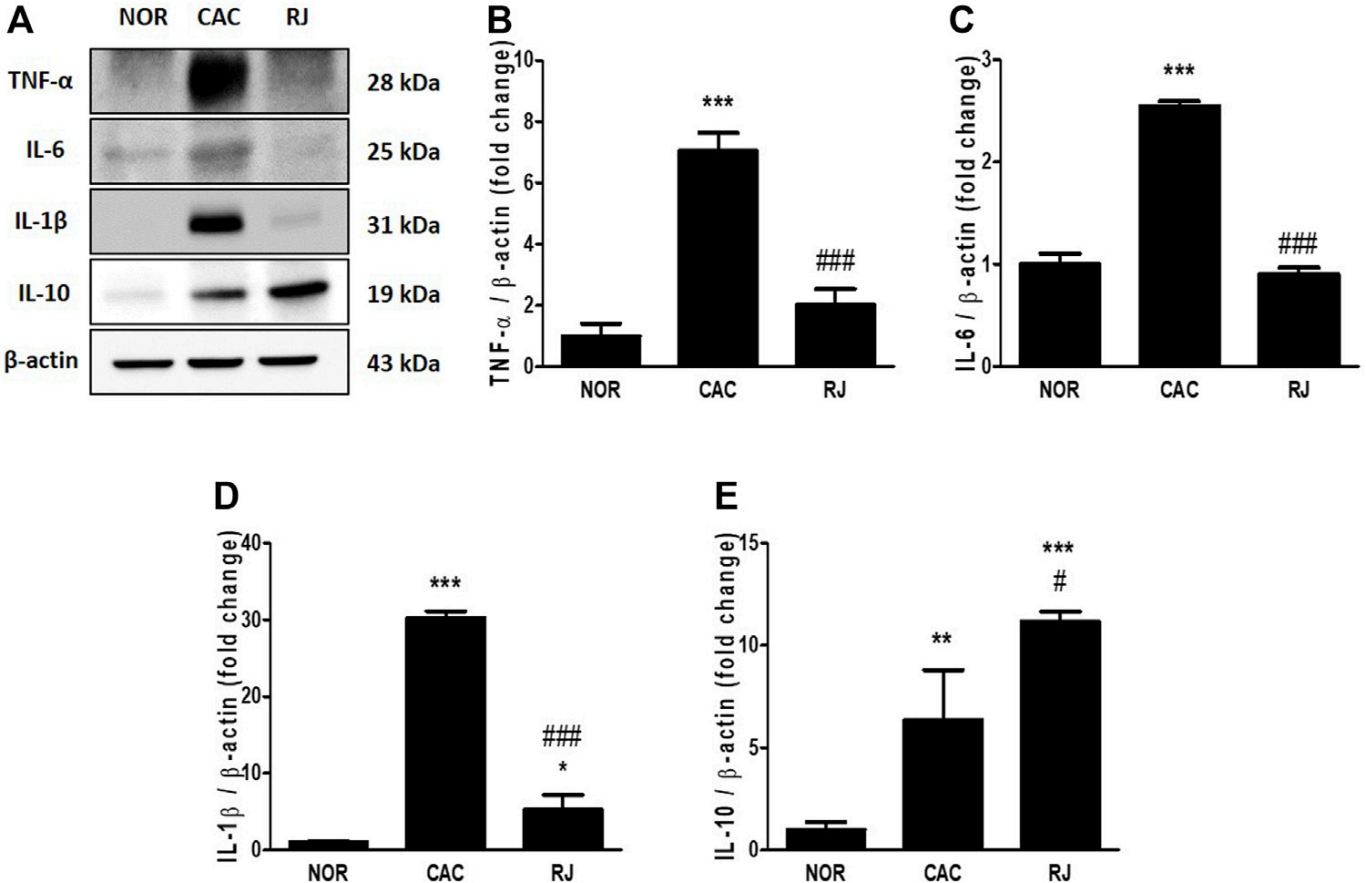
Regulatory effect of RJ on inflammatory cytokines in the colons of AOM/DSS-treated mice. By western blotting **(A)**, the protein expression levels of the pro- **(B–D)** or anti- **(E)** inflammatory cytokines in the colon were measured. Values are the means ± standard deviation (*n* = 3). ^*^ *p* < 0.05, ^**^ *p* < 0.01, and ^***^ *p* < 0.001 versus the normal group; #*p* < 0.01 and ^###^ *p* < 0.001 versus the CAC group. TNF, tumor necrosis factor; IL, interleukin.

**FIGURE 3:**
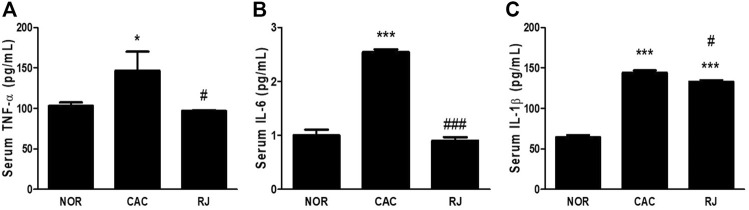
Regulatory effect of RJ on pro-inflammatory cytokines in the serum of AOM/DSS-treated mice **(A–C)**. Values are the means ± standard deviation (*n* = 3). ^*^ *p* < 0.05 and ^***^ *p* < 0.001 versus the normal group; ^#^ *p* < 0.01 and ^###^ *p* < 0.001 versus the CAC group. TNF, tumor necrosis factor; IL, interleukin.

In the CAC group, the levels of proinflammatory cytokines TNF-α, IL-6, and IL-1β were significantly higher than those in the normal group, but the levels in the RJ group were lower than those in the CAC group (Figures 2A–D). The level of the anti-inflammatory cytokine IL-10 was higher in the RJ group than that in the other groups (Figures 2A,E). In addition, changes in the levels of TNF-α, IL-6, and IL-1β (Figure 3) in the serum showed a tendency similar to that observed in the colon tissue.

### RJ Reduced the Collapse of TJs in the Colon of AOM/DSS-Induced Mice

**FIGURE 4:**
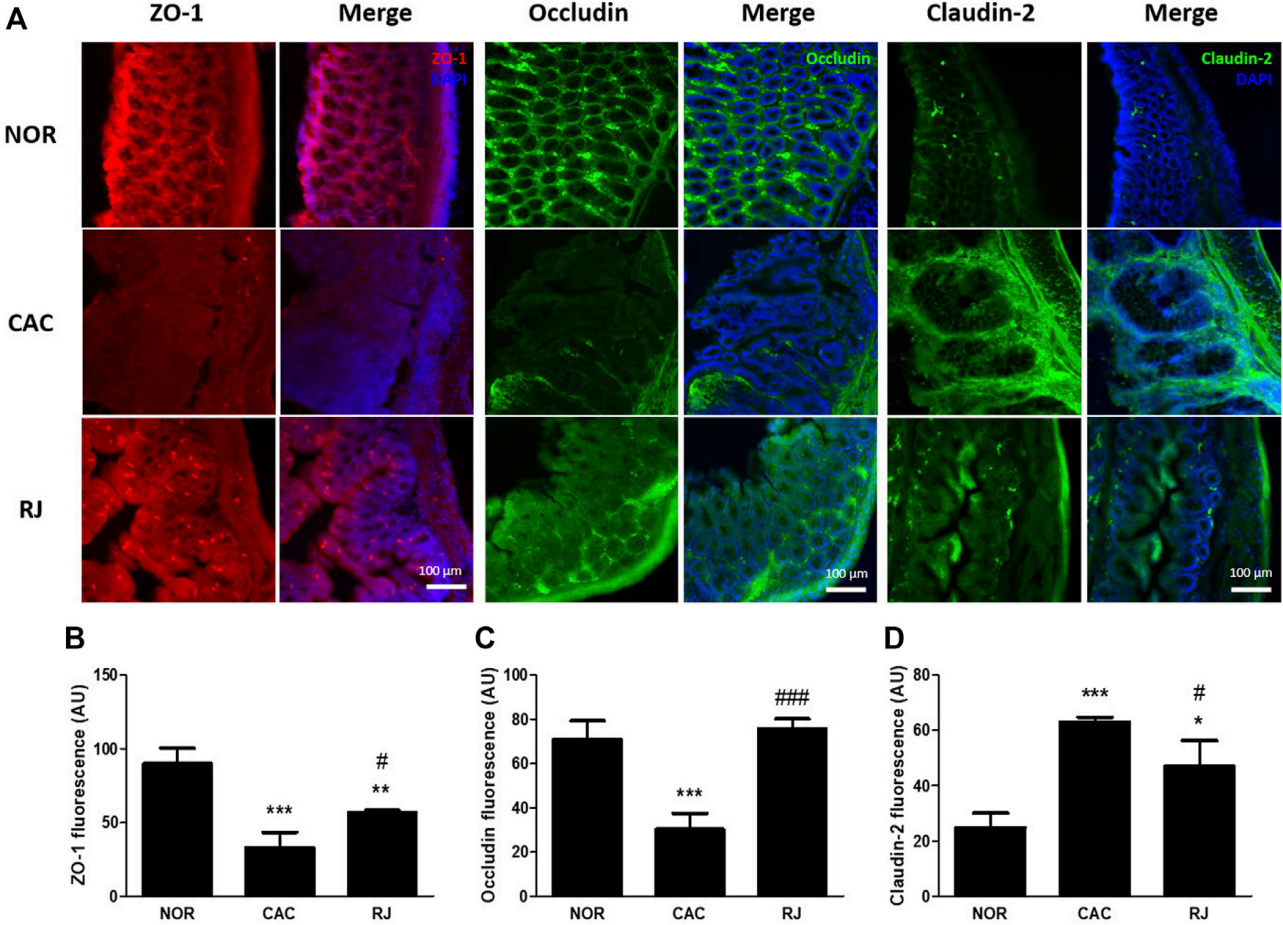
Protective effect of RJ on tight junctions in the colons of AOM/DSS-treated mice. Immunofluorescent pictures **(A)**, quantitative graphs **(B–D)** of ZO-1, occludin, and claudin-2 in the colon tissue are shown. Scale bar indicates 100 µm. Values are the means ± standard deviation (*n* = 3). ^*^ *p* < 0.05, ^**^ *p* < 0.01, and ^***^ *p* < 0.001 versus the normal group; ^#^ *p* < 0.05 and ^###^ *p* < 0.001 versus the CAC group.

**FIGURE 5:**
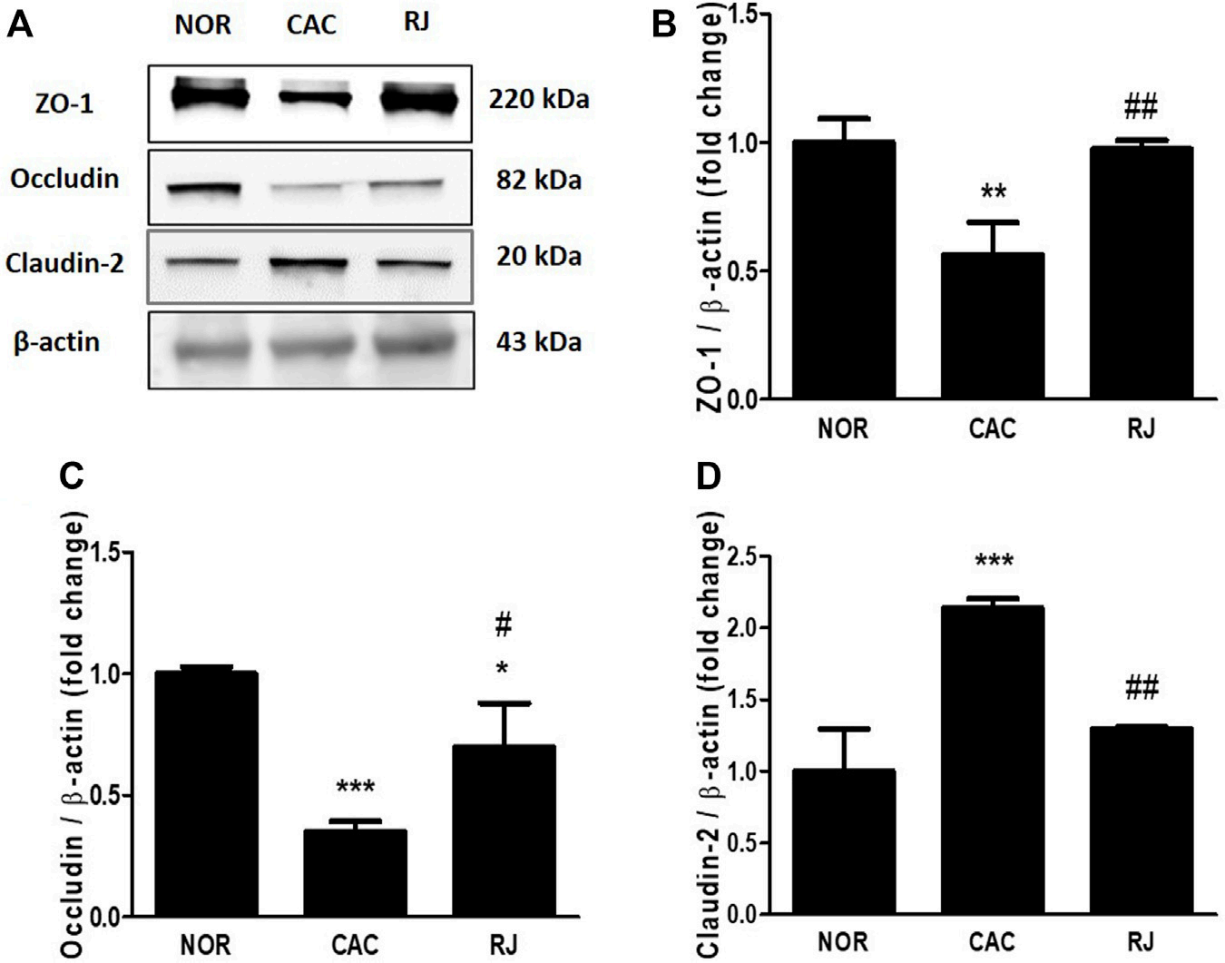
Protective effect of RJ on protein expression level of tight junctions in the colons of AOM/DSS-treated mice. By western blotting **(A)**, the levels of ZO-1 **(B)**, occludin **(C)**, and claudin-2 **(D)** were measured. Values are the means ± standard deviation (*n* = 3). ^*^ *p* < 0.05, ^**^ *p* < 0.01, and ^***^ *p* < 0.001 versus the normal group; ^#^ *p* < 0.05 and ^##^ *p* < 0.01 versus the CAC group.

The expression of TJ proteins in colon tissues was confirmed using immunofluorescence staining and western blotting. In the CAC group, the expression levels of ZO-1 and occludin were significantly reduced compared to those in the normal group, but this reduction was alleviated in the RJ group (F5–C, 5–C). AOM/DSS treatment significantly increased the claudin-2 expression level in the colon, which was inhibited by RJ treatment (Figures 4A,D, 5A,D).

### RJ Decreased Colonic Apoptosis in AOM/DSS-Induced Mice

**FIGURE 6:**
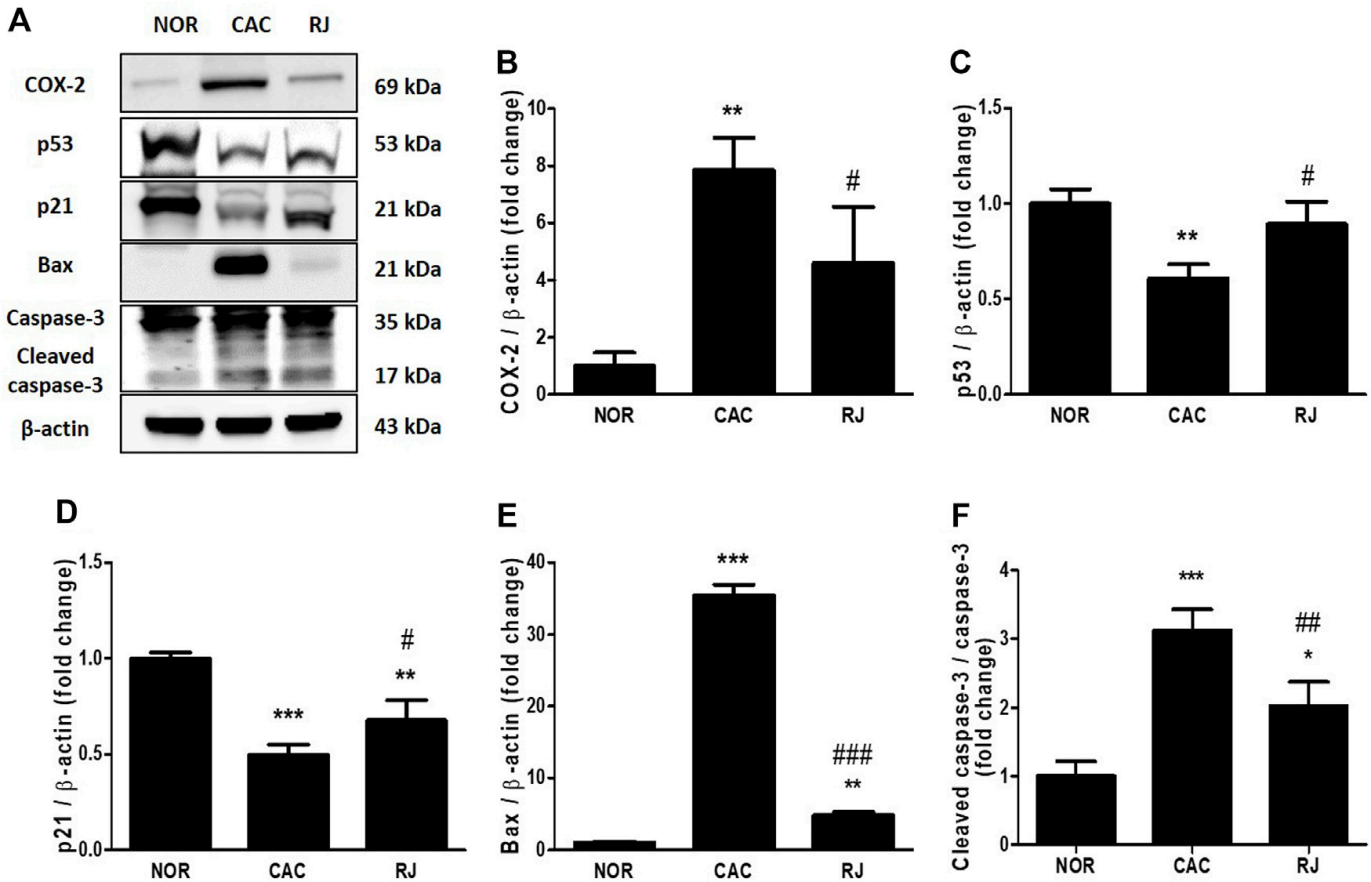
Inhibitory effect of RJ on AOM/DSS-induced apoptosis in the colons of mice. By western blotting **(A)**, the levels of COX-2 **(B)**, p53 **(C)**, p21 **(D)**, bax **(E)**, and caspase-3 **(F)** were measured. Values are the means ± standard deviation (*n* = 3). ^**^ *p* < 0.01 and ^***^ *p* < 0.001 versus the normal group; ^#^ *p* < 0.05 and ^###^ *p* < 0.001 versus the CAC group. COX, cyclooxygenase.

The expression levels of apoptosis-related proteins in the colon tissue are presented in Figure 6. Compared to the normal group, the CAC group showed an increase in COX-2 (*p* < 0.01), Bax (*p* < 0.001), and cleaved caspase-3 (*p* < 0.001), as well as decreased levels of p53 (*p* < 0.01) and p21 (*p* < 0.001). However, RJ treatment inhibited the increase in COX-2 (*p* < 0.05), Bax (*p* < 0.001), and cleaved caspase-3 (*p* < 0.001) levels, and decreased the levels of p53 (*p* < 0.05) and p21 (*p* < 0.05).

## Discussion

The AOM/DSS model is the most commonly used model of chemically induced colon carcinogenesis because the pathogenesis of the model is similar to human colorectal cancer (De Robertis et al., 2011). The combination of AOM (tumor-inducing agent) and DSS (tumor-promoting agent) shows synergistic effects to promote colorectal cancer in the initial acute inflammation phase (De Robertis et al., 2011). Treatment of AOM/DSS induces excremental change (diarrhea and bloody feces), shortens the colon length, and increases the weight and wall thickness of the colon because DSS causes inflammation (Suzuki et al., 2006). In the present study, AOM/DSS-treated mice exhibited shorter and heavier colons than normal mice, and contained many colon tumors. However, RJ treatment inhibited morphological changes in the colon (Figures 1B–D). Moreover, the mice in the CAC group defecated with severe bloody diarrhea or black feces (blood-containing stool), but the mice in the RJ group showed fewer defecatory problems (data not shown). These results indicated that RJ suppressed AOM/DSS-induced morphological and excremental changes in the colon.

Proinflammatory cytokines, including TNF-α, IL-6, and IL-1β, play an important role in triggering carcinogenesis (Xing et al., 2017). TNF-α and IL-6 are critical tumor promotors during tumorigenesis and colonic carcinogenesis in humans and mice (Yao et al., 2019). The active form of TNF-α was detected in the colon tissues in patients with UC and colorectal cancer but not in healthy individuals (Popivanova et al., 2008). In the AOM/DSS model, the activation of TNF-α has been demonstrated to be caused by DSS rather than AOM, and TNF-α accelerates progression of colon carcinomas by increasing COX-2 expression level and inhibiting the Wnt signaling pathway (Popivanova et al., 2008). In patients with IBD or colorectal cancer, the levels of circulating and colonic IL-6 were increased, which were also found in the DSS- or AOM/DSS-treated mice (Yao et al., 2019). Also, IL-6 knockout mice treated with AOM/DSS formed fewer tumors and smaller adenomas and tumors, suggesting that IL-6 is important to the formation and growth of tumors in CAC (Grivennikov et al., 2009). In addition, TNF-α and IL-6 can affect each other; thus, the cross-regulation of these proinflammatory cytokines may be involved in CAC development to enhance inflammation and tumorigenesis (Grivennikov et al., 2009; Yao et al., 2019). The mice deficient in the anti-inflammatory cytokine IL-10 showed spontaneous gut inflammation (Sato et al., 2006), and the treatment of IL-10 decreased the level of TNF-α, IL-6, and IL-1β in an IBD animal model (Li and He, 2004). Therefore, pro- and anti-inflammatory cytokines play a critical role in inflammation and tumorigenesis in the colon. In the present study, treatment with RJ effectively regulated cytokines TNF-α, IL-6, IL-1β, and IL-10, which may contribute to alleviating the pathological changes caused by AOM/DSS in the colon (Figures 1E, 2, 3).

The intestinal barrier maintains homeostasis by inhibiting the penetration of pathogens or toxic substrates while allowing the absorption of nutrients into the intestine (Landy et al., 2016). TJs, the major components of the intestinal epithelial barrier, construct the TJ barrier by connecting neighboring epithelial cells and controlling paracellular transport (Xing et al., 2017). TJs are composed of occludin, claudins, ZO proteins, junctional adhesion molecules, tricellulin, and cingulin (Landy et al., 2016). The transmembrane proteins, occludin and claudin, are the most important controllers of gut barrier function, and the framework element ZO proteins form the central network for protein interactions (Xing et al., 2017). Occludin supports the structure of the TJ barrier and regulates paracellular permeability by binding to ZO-1 (Xing et al., 2017). Among the 27 isoforms of claudin, claudin-1 and -2 are expressed in leaky gut with inflammation (Xing et al., 2017). In IBD and colorectal cancer, the expression levels of occludin and ZO-1 are reduced, but the expression level of claudin-2 is increased (Landy et al., 2016). Claudin-2 expression level can be increased by the activation of IL-6 (Suzuki et al., 2011), and increased claudin-2 level enhances IBD-associated dysplasia and sporadic adenomas (Weber et al., 2008). When the connection of TJs is weak, the immune cells move to the intestine through the paracellular pathway and produce proinflammatory cytokines, such as TNF-α, IL-6, and IL-1β (Kim et al., 2022). Therefore, the weakness of the TJ barrier causes epithelial inflammatory damage, such as erosion, ulceration, and apoptosis, which are major characteristics of IBD and colorectal cancer (Landy et al., 2016). In the present study, treatment of RJ effectively protected the TJ barrier and suppressed the activation of cancerous TJ like claudin-2 in the colon tissue (Figures 4, 5). In addition, in our previous studies, RJ protected the TJ barrier not only in the colon but also in the brain (Kim et al., 2020; Kim et al., 2022). Therefore, RJ may enhance the connection between TJs.

The self-renewal of intestinal epithelial cells (IECs) is important for maintaining the homeostasis of the colon, but the process cannot work properly under inflammatory conditions (Yao et al., 2019). Consequentially, inflammation and apoptosis of IECs negatively affect the normal function of the gut barrier. In fact, the inflamed sigmoid colon tissue from UC patients leaks from apoptotic foci, which may worsen depending on the severity of the inflammation (Gitter et al., 2001). A cytoplasmic protein, COX-2, catalyzes the synthesis of lipid inflammatory substrates, such as prostaglandin from arachidonic acid, and contributes to tumorigenesis by enhancing apoptosis, angiogenesis, and invasiveness of cancer cells (De Robertis et al., 2011). According to Eberhart et al., COX-2 is increased in the inflammatory area in ∼80% of colorectal cancers and 40% of colorectal adenomas (Eberhart et al., 1994). The tumor suppressor p53 plays a role in cell cycle arrest and apoptosis in the development of colorectal cancer neoplasm (De Robertis et al., 2011). The protein p53 regulates p21, which is involved in permanent and transient cell cycle arrest by suppressing the activity of cyclin-dependent kinases, preventing cell cycle transition from G1 to S phase (Pitolli et al., 2019). In addition, p53 regulates Bax, an apoptosis mediator (Pitolli et al., 2019). Caspase-3 is activated at the final step of the caspase cascade in apoptosis. In the present study, treatment with RJ regulated the expression of apoptosis-related proteins in the colon tissues of AOM/DSS-treated mice (Figure 6), which is similar to the results of our previous studies on the effect of RJ in colitis and Parkinson’s disease mouse models (Kim et al., 2020; Kim et al., 2022). These results indicate that RJ has anti-inflammatory and anti-apoptotic effects that contribute to the suppression of CAC.

This study had a few limitations. First, RJ contains various substances, and it is unclear which substances are responsible for the therapeutic effect. Second, the anti-cancer and protective effects of RJ in the colon need to be verified through clinical trials. Further studies are required to confirm these results.

In the present study, we demonstrated that RJ can protect the colon by inhibiting inflammation, TJ barrier disruption, and apoptosis in CAC mice. Moreover, RJ decreased the formation of colonic tumors. Therefore, RJ has potential for use in treating colon diseases, such as colitis, colorectal cancer, and diseases related to the intestinal immune response.

## Data Availability

The original contributions presented in the study are included in the article/Supplementary Material, further inquiries can be directed to the corresponding author.
