# Supplementary material for: Rumex japonicus Houtt. Extract Suppresses Colitis-Associated Colorectal Cancer by Regulating Inflammation and Tight-Junction Integrity in Mice
Source: Front Pharmacol. 2022 Jul 5;13:946909. doi: 10.3389/fphar.2022.946909 (PMC9294457; doi:10.3389/fphar.2022.946909)
Supplement: Supplementary file 1 [file DataSheet1.PDF]

Supplementary material

Rumex japonicus Houtt. Extract Suppresses Colitis-associated Colorectal Cancer by Regulating Inflammation and Tight-junction Integrity in Mice

Short running title: Effect of *Rumex japonicus* Houtt. on Colorectal Cancer

Hee-Young Kim[1], Ji Eun Seo[2], Hanul Lee[2], Chang Hwan Bae[2], Ki-Tae Ha[1,2], Seungtae Kim[1,2,*]

[1]Korean Medicine Research Center for Healthy Aging, Pusan National University, Yangsan, Republic of Korea

[2]Department of Korean Medical Science, School of Korean Medicine, Pusan National University, Yangsan, Republic of Korea

***Correspondence:** Prof. Seungtae Kim

kimst@pusan.ac.kr

SUPPLEMENTARY METHODS

Ultra-Performance Liquid Chromatography Coupled with Quadrupole-Time-Of-Flight Tandem Mass Spectrometry (UPLC-Q-TOF-MS) Analysis

UPLC-Q-TOF-MS analysis using an Agilent 1290 Infinity LC system coupled with an Agilent 6530 Accurate-Mass-Q-TOF-LC/MS (Agilent Technologies, Santa Clara, CA, USA) was performed to confirm anthraquinones contained in the RJ extract. Chromatographic separations were performed using an ACQUITY BEH C18 column (2.1 mm × 100 mm, 1.7 μm, Waters, Milford, Ireland) maintained at 40 ℃. The mobile phase consisted of 0.1% (v/v) formic acid and acetonitrile (solvent B) with a gradient program: 0–1.5 min, 0–10% B; 1.5–6 min, 10–17% B; 6–12 min, 17–43% B; 12–21 min, 43–95%; 21–21.1 min, 95–5% B; 21.1–25 min, 5% B; and maintained at 5% until stop. The flow rate was 0.3 mL/min, and the injection volume was 10 μL. The mass spectra were acquired in negative mode, m/z ranging from 100 to 1300, with electrospray ionization (ESI). The optimized MS conditions were as follows: gas flow 9 L/min, gas temperature 300 ℃, nebulizer 45 psi, capillary voltage 4000 V, skimmer voltage 65 V, octopole RF voltage 750 V, fragmentor voltage 175 V. The obtained mass spectra were processed using Acquisition Software Version, 6200 se-ries TOF/6500 series Q-TOF B.05.01 (B5125.1). The components were identified based on chromatographic retention time, formulation composition, references, and data-base PubChem [available online: https://pubchem.ncbi.nlm.nih.gov/ (accessed on 26 October 2021)].

SUPPLEMENTARY FIGURES

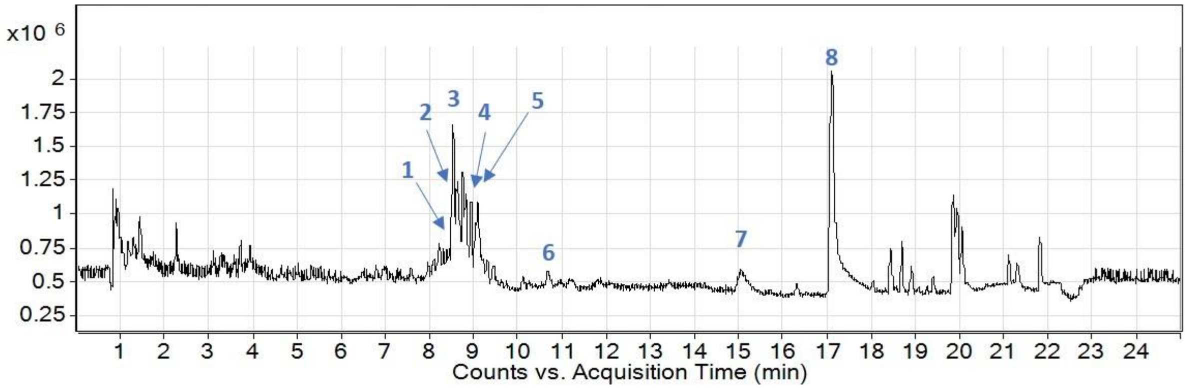

No.	RT (Min)	$[M-H]^-$ Molecular Formula	$[M-H]^-$ (m/z) Measured	Predicted Identity
1	8.612	$C_{21}H_{20}O_{10}$	431.0987	Emodin-8-glucoside
2	8.706	$C_{21}H_{20}O_9$	415.1030	Chrysophanol-8-O-β-D-glucoside (pulmatin)
3	8.756	$C_{15}H_8O_6$	283.0255	Rhein
4	9.022	$C_{15}H_{10}O_4$	253.0505	Chrysophanol
5	9.049	$C_{22}H_{22}O_{10}$	445.1140	Physcion-8-O-β-D-glucopyranoside
6	10.840	$C_{16}H_{12}O_5$	283.0612	Physcion
7	15.058	$C_{15}H_{10}O_5$	269.0458	Aloe emodin
8	17.098	$C_{15}H_{10}O_5$	269.0456	Emodin

Supplementary Figure S1. Constituents of RJ extract identified by UPLC-Q-TOF-MS.

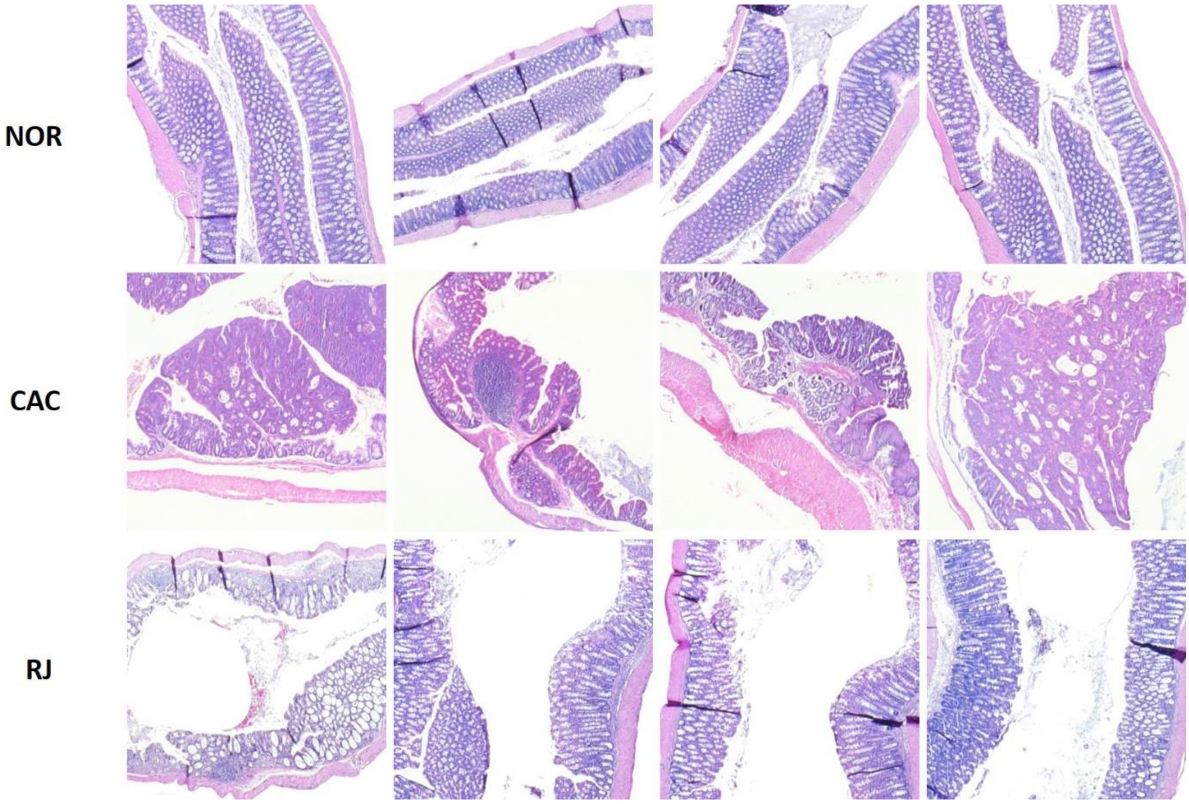

Supplementary Figure S2. Histological changes in the colons of the azoxymethane and dextran sulfate sodium- or RJ-treated mice (25×; n = 4 in each group).
